# Fibrocalculous pancreatic diabetes in a young Ugandan patient, a rare form of secondary diabetes

**DOI:** 10.1186/1756-0500-5-622

**Published:** 2012-11-05

**Authors:** Davis Kibirige, Solomon Kibudde, Edrisa Mutebi

**Affiliations:** 1 Department of Medicine, Makerere University College of Health Sciences and Endocrine unit, Mulago national referral and teaching Hospital, P.O.BOX 7062, Kampala, Uganda

**Keywords:** Fibrocalculous pancreatic diabetes, Tropical diabetes, Chronic pancreatitis, Secondary diabetes, Uganda

## Abstract

**Background:**

Fibrocalculous pancreatic diabetes is an infrequent type of secondary diabetes due to chronic tropical non alcoholic calcific pancreatitis. It has been widely described exclusively in developing tropical countries. A diagnosis is made basing on the presence of abdominal pain, presence of pancreatic calcifications, steatorrhoea, and diabetes mellitus.

**Case presentation:**

We report a case of a 20 year old Ugandan female patient who presented with features of chronic tropical calcific pancreatitis complicated by diabetes mellitus, oedematous malnutrition and micronutrient deficiencies.

**Conclusion:**

This case report demonstrates that fibrocalculous pancreatic diabetes still exists in developing countries like Uganda. Clinicians in such settings should possess a high clinical suspicion of fibrocalculous pancreatic diabetes especially in presence of malnutrition. Challenges of management of such patients in resource limited settings are comprehensively discussed in the review of literature.

## Background

In addition to the classical type 1 and type 2 forms of diabetes, other rare forms of diabetes mellitus (DM) like fibrocalculous pancreatic diabetes (FPD) have been described and still exist in SSA [1].

Tropical calcific pancreatitis (TCP) is a juvenile form of chronic calcific, non alcoholic pancreatitis highly prevalent in developing and tropical countries. It is often associated with pancreatic exocrine and endocrine dysfunction. With occurrence of DM, it is referred to as FPD. It develops as a late complication of TCP and it is often very severe [2].

Several cases reports of FPD have been extensively described in literature from tropical, poverty stricken African [3,4] and Asian [5,6] countries for over 50 years.

## Case presentation

A 20 year old female patient was referred from a rural hospital to the endocrine unit with a three year history of generalised body weakness associated with progressive weight loss and recurrent generalised abdominal pain. She also had a five month history of polydipsia, polyuria and a day’s history of high grade fever with dysuria. She had no history of steatorrhoea.

Prior to her referral, she was being treated as a patient with type 1 DM for 4 months in a rural hospital. She also received analgesics and multi vitamins as treatment for the generalised abdominal pain and body weakness respectively. However, due to inadequate resources at that rural hospital, no specific clinical investigation was done to determine the cause of her recurrent abdominal pain. Her HIV serology was negative.

She was the 5^th^ child of seven and all her siblings were healthy. There was no familial history of diabetes. She had no history of alcohol ingestion. Her diet since childhood was predominantly rich in carbohydrates.

Physical examination revealed a young lady with a low body mass index of 15.8kg/m^2^. She had sparse silky hair with bilateral cataracts, mild pallor of the mucous membranes, atrophic glossitis, leuconychia and bilateral pedal oedema. No skin changes or any bleeding tendencies were noted.

On the neurological examination, she was fully conscious but appeared apathetic. She had a slow thought process and poor short-term memory. Deep tendon reflexes, joint position and vibration senses were not assessesed because the patient was very unco-operative. Musculoskeletal examination revealed generalised muscle atrophy with tenderness of the bones and over the spine vertebrae.

At presentation to the endocrine unit, the haematological investigations done included a raised random blood sugar level of 26.7 mmol/l (normal: 3.5-7.7). The complete blood count showed a leucocytosis of 21,900/mm^3^ (normal: 4,000-10,000), mild normocytic normochromic anemia of 10.8 g/dl (normal: 12–16) and a thrombocytopenia of 60,000/mm^3^ (normal: 150,000-400,000). She had severe hypoalbuminemia of 18.9g/dl (normal: 35–50) and a corrected hypocalcaemia of 7.2 mg/dl (normal: 9–10.5 mg/dl). The serum vitamin B12 levels were reduced (128.9 pg/ml [normal range: 204–900]). The lipid profile, renal and liver function tests were normal. The urinalysis showed glycosuria, numerous pus cells, mild proteinuria but no ketonuria. The stool analysis revealed no fat globules, ova and cysts. However, this was done without prior fat loading.

Assessment of the glycated haemoglobin (HbA1c) and fasting C-peptide levels were not done because of the high costs involved and were unavailable at the hospital at the time of the patient’s admission.

**Figure 1:**
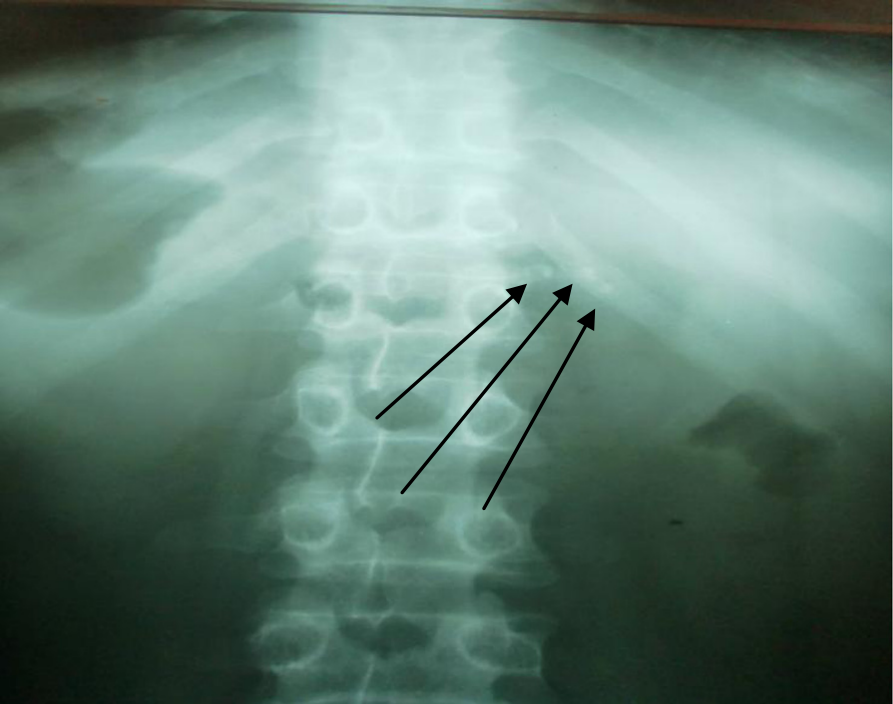
**Erect plain X-ray showing pancreatic calcifications. **The arrows illustrate the calcifications in the pancreatic region.

The abdominal X-ray showed multiple pancreatic calcifications (Figure 1). An abdominal ultrasound scan showed diffuse calcifications in the head, body, and tail of the pancreas.

A diagnosis of FPD with severe oedematous malnutrition and micronutrient deficiency complicated by a urological sepsis was made.

The patient achieved good glycemic control on pre-mixed insulin (Mixtard) in small doses of 10IU pre breakfast and 5IU pre supper. She also received pancreatic enzyme granules given with meals and the co-existing micro nutrient deficiencies were corrected with vitamin A, D, B_12_ and calcium replacement. The urinary tract infection was treated with oral ciprofloxacin.

She was adequately counselled on the features of both hypoglycemia and hyperglycemia since she could not afford a glucometer to do regular self blood glucose monitoring. She also received dietary counselling with emphasis on a high protein diet. She was discharged when she had greatly improved. All serial fasting blood glucose levels done prior to discharge were within the normal ranges. Unfortunately, she has been lost to follow-up because she could not afford the transport costs needed for the clinic reviews.

## Discussion

In this case report, we report a case of a young African patient presenting with clinical features of FPD. Similar presentation has been described in the documented cases reported in other parts of Africa [3,4] and Asia [5,6].

The classical recognised clinical picture of FPD is primarily of a young diabetic patient presenting with recurrent epigastric pain, steatorrhoea, signs of malnutrition and micro nutrient deficiencies. Steatorrhoea is however, relatively rare especially in areas with low dietary fat intake [7] as demonstrated in this case report.

Multiple factors have been thought to be associated with FPD. These include malnutrition; toxic effects of cyanide derived from frequent cassava consumption, familial aggregation, genetic factors and increased oxidant stress from micronutrient deficiency (vitamin C and A deficiencies) [7,8].

However, several recent reports have disputed the theories that malnutrition and frequent cassava ingestion have aetiological roles in FCP. Studies from certain regions of the world with very high frequencies of malnutrition like Ethiopia have documented a relatively low frequency of FCP [9]. A study by Swai et al. compared two rural Tanzanian populations of which one had cassava as the staple food and also elevated urinary and plasma cyanide levels and noted no significant difference in glucose tolerance or magnitude of DM [10].

Genetic factors have been proposed as the most significant in the aetiology of FCP. Current evidence has confirmed a link between the serine protease inhibitor, Kazal type 1 (SPINK 1) gene and TCP [11,12]. It is a vital protease inhibitor that prevents unregulated or inappropriate activation of the pancreatic enzyme cascade by inhibiting trypsin activity [13]. This would eventually result into recurrent pancreatitis.

Demonstration of hyperglycemia and pancreatic calculi on plain abdominal X ray, abdominal computed tomography scan or ultrasound scan confirms the diagnosis as shown in the patient discussed [14].

Patients with FCP usually require treatment with insulin though in small quantities. This is due to the presence of residual beta cell function reflected by the intermediate C- peptide levels among these patients [15]. As demonstrated in the case report, ketosis among patients with FPD is very infrequent. This is because of the residual pancreatic beta-cell reserve, a low glucagon reserve and decreased adipose tissue mass [15].

The main challenge involved in the management of patients with FPD in resource limited countries is recurrent episodes of hypoglycemia. This is due to the partial preservation of the pancreatic beta cell mass and function and lack of continuous self monitoring of blood glucose among these patients because of related costs involved. This results into increased morbidity and mortality. Delayed or misdiagnosis of the cases, inadequate diabetic medicines especially insulin, protracted micronutrient deficiencies and inability to adequately correct micro and macro nutrient deficiency are also other management challenges.

## Conclusion

FPD still exists in developing countries. An active approach in recognising such cases should be adopted in these settings. Conservative glycemic control, frequent blood glucose monitoring and correction of the micro and macronutrient deficiencies are very fundamental in the management of patients with FPD and should be strongly emphasised.

## Consent

Written informed consent was obtained from the patient for publication of this case report and any accompanying images. A copy of the written consent is available for review by the Editor-in-Chief of this journal.

## Competing interests

The authors declare that they have no competing interests.

## Authors’ contributions

DK participated in the care of the patient in the endocrine unit and drafted the manuscript. SK and EM also participated in the care of the patient in the endocrinology unit and critically revised the contents of the manuscript. All authors approved the final draft of the manuscript for submission for publication.
